# Gut Microbiome and Serum Metabolome Alterations Associated with Isolated Dystonia

**DOI:** 10.1128/mSphere.00283-21

**Published:** 2021-08-04

**Authors:** Lingyan Ma, Jing Keng, Min Cheng, Hua Pan, Bo Feng, Yongfeng Hu, Tao Feng, Fan Yang

**Affiliations:** a Center for Movement Disorders, Department of Neurology, Beijing Tiantan Hospital, Capital Medical University, Beijing, China; b NHC Key Laboratory of Systems Biology of Pathogens, Chinese Academy of Medical Sciences (CAMS) & Peking Union Medical College (PUMC), Beijing, China; c China National Clinical Research Center for Neurological Diseases, Beijing, China; d China Institute of Veterinary Drug Control, Beijing, China; e Parkinson's Disease Center, Beijing Institute for Brain Disorders, Beijing, China; f Department of Neurology, Beijing Tiantan Hospital, Capital Medical University, Beijing, China; g Safe Transfusion Lab, Beijing Red Cross Blood Center, Beijing, China; University of Michigan-Ann Arbor

**Keywords:** dystonia, metagenomics, 16S rRNA, gut microbiome, serum metabolome

## Abstract

Dystonia is a complex neurological movement disorder characterized by involuntary muscle contractions. Increasing studies implicate the microbiome as a possible key susceptibility factor for neurological disorders, but the relationship between the gut microbiota and dystonia remains poorly explored. Here, the gut microbiota of 57 patients with isolated dystonia and 27 age- and environment-matched healthy controls was analyzed by 16S rRNA gene amplicon sequencing. Further, integrative analysis of the gut microbiome and serum metabolome measured by high-performance liquid chromatography-mass spectrometry was performed. No difference in α-diversity was found, while β-diversity was significantly different, with a more heterogeneous community structure among dystonia patients than among controls. The most significant changes in dystonia highlighted an increase in *Clostridiales*, including Blautia obeum, Dorea longicatena, and Eubacterium hallii, and a reduction in Bacteroides vulgatus and Bacteroides plebeius. The functional analysis revealed that genes related to tryptophan and purine biosynthesis were more abundant in gut microbiota from patients with dystonia, while genes linked to citrate cycle, vitamin B_6_, and glycan metabolism were less abundant. The evaluation of serum metabolites revealed altered levels of l-glutamic acid, taurine, and d-tyrosine, suggesting changes in neurotransmitter metabolism. The most modified metabolites strongly inversely correlated with the abundance of members belonging to the *Clostridiales*, revealing the effect of the gut microbiota on neurometabolic activity. This study is the first to reveal gut microbial dysbiosis in patients with isolated dystonia and identified potential links between gut microbiota and serum neurotransmitters, providing new insight into the pathogenesis of isolated dystonia.

**IMPORTANCE** Dystonia is the third most common movement disorder after essential tremor and Parkinson’s disease. However, the cause for the majority of cases is not known. This is the first study so far that reveals significant alterations of gut microbiome and correlates the alteration of serum metabolites with gut dysbiosis in patients with isolated dystonia. We demonstrated a general overrepresentation of *Clostridiales* and underrepresentation of *Bacteroidetes* in patients with dystonia in comparison with healthy controls. The functional analysis found that genes related to the biosynthesis of tryptophan, which is the precursor of the neurotransmitter serotonin, were more active in isolated dystonia patients. Altered levels of several serum metabolites were found to be associated with microbial changes, such as d-tyrosine, taurine, and glutamate, indicating differences in neurotransmitter metabolism in isolated dystonia. Integrative analysis suggests that neurotransmitter system dysfunction may be a possible pathway by which the gut microbiome participates in the development of dystonia. The gut microbiome changes provide new insight into the pathogenesis of dystonia, suggesting new potential therapeutic directions.

## INTRODUCTION

Dystonia is a complex, highly variable neurological movement disorder characterized by sustained or intermittent muscle contractions (1). It can be the manifestation of many neurological disorders, either in isolation (isolated dystonia) or in combination with additional symptoms (combined dystonia) (2). Isolated dystonia includes syndromes in which dystonia is the sole phenotypic manifestation, with the exception of tremor (1). Until now, most patients with common adult-onset dystonia tend to arise sporadically with no clear etiology and pathogenesis, though several associated genes (*TOR1A*, *THAP1*, *GCH1*, and *KMT2B*) have been identified as the cause of childhood- and adolescent-onset dystonia (3), and these mechanisms include abnormalities in transcriptional regulation, striatal dopaminergic signaling and synaptic plasticity, and a loss of inhibition at neuronal circuits. Clinical electrophysiological studies showed that the dystonic central nervous system (CNS) exhibits to some extent deficient inhibitory neurotransmission, and dystonia is more likely a disorder of motor circuits in a particular brain structure. Researchers believe that dystonia results from an abnormality in the basal ganglia circuits or other brain regions that control movement and, possibly, abnormalities in processing a group of chemicals called neurotransmitters, such as dopamine, serotonin (5-HT), gamma-aminobutyrate (GABA), acetylcholine, purine, and norepinephrine, which help cells in the brain communicate with each other (4, 5).

The gut microbiome contains a complex community of microbes that live within the gastrointestinal tract. The brain-gut-microbiome axis allows the brain to control gut function but also provides an opportunity for the gut microbiome to influence the brain (6). Emerging evidence has implicated the gut microbiome as an important player in neurodevelopment, as well as in brain diseases such as Parkinson's disease (PD) (7), Alzheimer’s disease (AD) (8), and multiple sclerosis (9). Gut microbes communicate with the central nervous system through nervous-, endocrine-, and immune-signaling mechanisms. As being increasingly recognized, gut permeability, bacterial metabolites (such as short-chain fatty acids [SCFA] and lipopolysaccharide [LPS]), neurotransmitters (e.g., GABA, dopamine, and serotonin), and the vagal nerve are perhaps the most important factors in initiating and mediating microbial interactions with the central nervous system of the body. It has been reported that anticlostridial agents were capable of reversing both gastrointestinal and neurological symptoms in one sporadic case of myoclonus-dystonia (10), providing clues to the mechanisms driving dystonia and potentially other neurological conditions with a gut microbial component. However, the relationship between the gut microbiota and isolated dystonia remains poorly explored.

Using 16S rRNA gene amplicon and metagenomic sequencing, we investigated gut microbial characteristics in isolated dystonia and found that dystonia patients carry an altered gut microbiota enriched with Eubacterium hallii, Blautia obeum, and Dorea longicatena. Functional analyses also suggested differences in microbial tyrosine and tryptophan biosynthesis, purine metabolism, and sulfur metabolism pathways. We further performed integrative analysis of gut metagenome and nontargeted serum metabolome profiling of patients with significant gut dysbiosis and revealed an association of the altered gut microbiome with serum metabolites (especially neurotransmitters) in the host.

## RESULTS

### Demographics and clinical data.

Fifty-seven isolated dystonia patients and 27 healthy controls (HCs) were recruited, and an integrative analysis of the gut microbiome and serum metabolome was performed. Clinical details of the study cohort are shown in Table 1.

**TABLE 1:** Demographics of subjects

Parameter^*a*^	Value for:		*P* value
	Dystonia patients (*n* = 57)	HCs (*n* = 27)	
No. (%) of males	15 (26.32)	9 (33.33)	0.506
No. (%) of females	42 (73.68)	18 (66.67)	
BMI (mean ± SD)	22.71 ± 2.04	23.61 ± 1.80	0.341
Age (yr) (mean ± SD)	45.25 ± 13.00	41.1 ± 11.15	0.158
Age at onset (yr) (mean ± SD)	45.05 ± 12.86		
Duration (mo) (mean ± SD)	5.32 ± 3.77		
UDRS score (mean ± SD)	9.91 ± 14.43		
GDS score (mean ± SD)	6.54 ± 4.06		
No. (%) with family history (*n*; %)	0		
HAMA score (mean ± SD)	8.51 ± 4.21	6.78 ± 3.11	0.060
HAMD score (mean ± SD)	8.14 ± 4.13	6.52 ± 2.67	0.066

a UDRS, Unified Dystonia Rating Scale; GDS, Global Dystonia Rating Scale; HAMA, Hamilton Anxiety Scale; HAMD, Hamilton Depression Scale.

### Data overview.

In total, 4,397,348 high-quality 16S rRNA gene V4 sequences were obtained, with 52,349 ± 8,462 (mean ± standard deviation [SD]) per sample after demultiplexing and quality control (see Table S1 in the supplemental material). A total of 997 operational taxonomic units (OTUs) (>97% similarity, excluding singletons and low-abundance taxa with a frequency of <3 for samples; range, 204 to 461; median, 306 taxa per sample) were identified, representing 22 taxonomic phyla. The dominant phyla were *Firmicutes* (66.4%), *Bacteroidetes* (17.5%), *Proteobacteria* (9.4%), and *Actinobacteria* (5.3%). At the genus level, *Bacteroides* (13%), *Faecalibacterium* (10%), unidentified *Lachnospiraceae* (7%), *Agathobacter* (6%), and *Blautia* (5%) were the most abundant, of which all but *Bacteroides* belong to *Clostridiales* in *Firmicutes* (Fig. S1).

10.1128/mSphere.00283-21.2 TABLE S1 Detailed data information obtained by 16S rRNA gene amplicon sequencing for the studied dystonia patients and HCs. Download Table S1, XLSX file, 0.6 MB. Copyright © 2021 Ma et al. 2021 Ma et al. https://creativecommons.org/licenses/by/4.0/ This content is distributed under the terms of the Creative Commons Attribution 4.0 International license.

10.1128/mSphere.00283-21.7 FIG S1 Bacterial composition of fecal samples collected from 57 isolated dystonia patients and 27 healthy controls and the relative abundance of the most abundant phyla and genera (representing 98.6% of the total reads). Download FIG S1, TIF file, 0.6 MB. Copyright © 2021 Ma et al. 2021 Ma et al. https://creativecommons.org/licenses/by/4.0/ This content is distributed under the terms of the Creative Commons Attribution 4.0 International license.

### Gut microbiota revealed by 16S rRNA gene amplicon sequencing.

**FIG 1:**
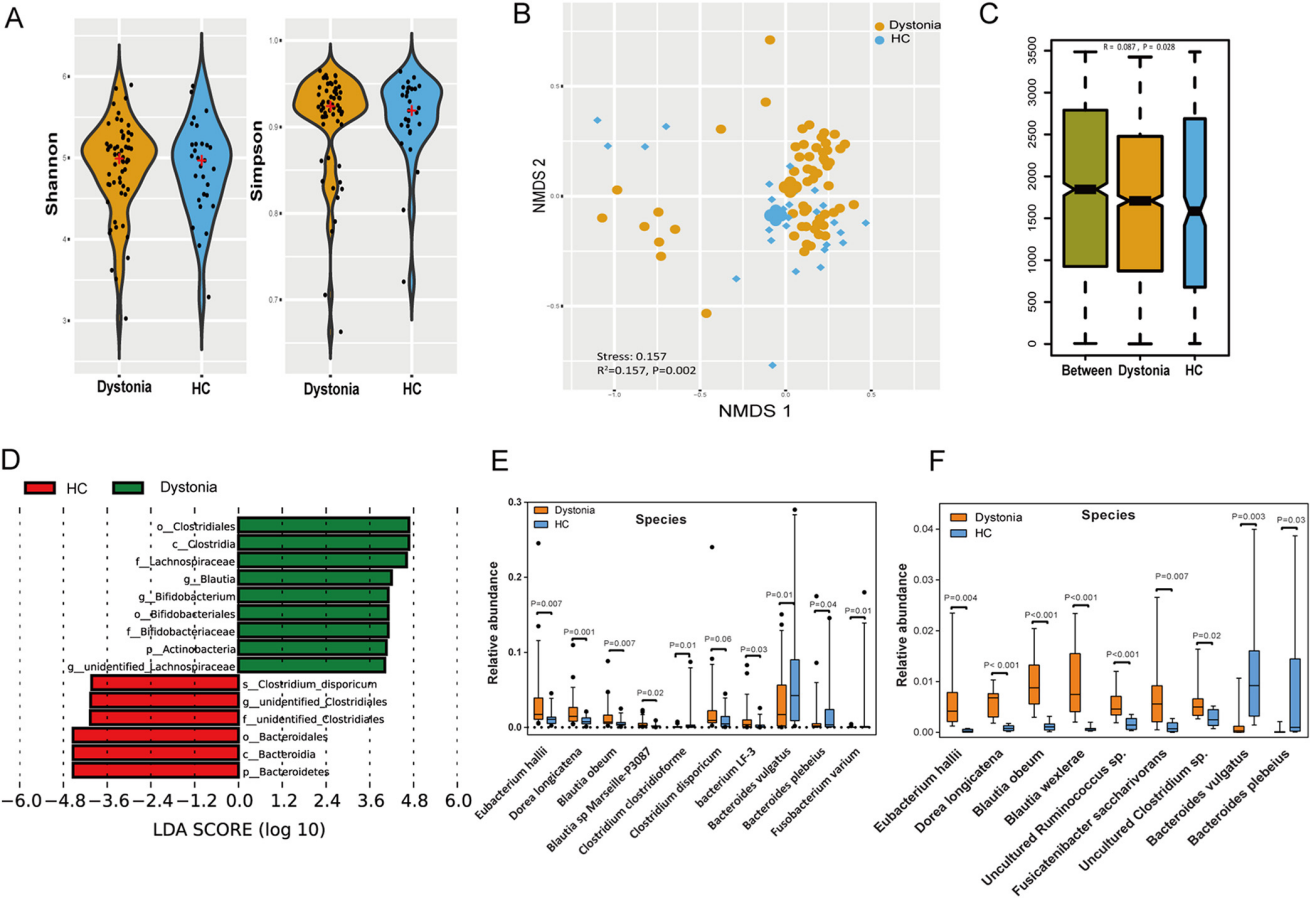
Gut microbiome differences between dystonia patients and HCs. (A) Alpha-diversity calculated by Shannon and Simpson indices. Significance was confirmed using Mann-Whitney U tests. (B) Beta-diversity of gut bacterial communities between isolated dystonia patients and HCs using unsupervised ordination (NMDS). Significant differences were confirmed by PERMANOVA (*R*^2^ = 0.157, *P = *0.002). Centroids are indicated by crosses. (C) Within-group and between-group dissimilarities analyzed by ANOSIM (*R* = 0.087, *P = *0.028). (D) LEfSe analysis revealing significant taxonomic differences between isolated dystonia patients and HCs. LDA, linear discriminant analysis. (E) Comparison of the relative abundances of the most abundant taxa (≥1%) at the species level in 16S rRNA gene amplicon sequencing. (F) Comparison of the relative abundances of the most abundant taxa (≥1%) at the species level in metagenomic sequencing.

Gut microbial α-diversity was not significantly different as calculated by Shannon and Simpson indices (Fig. 1A), while nonmetric multidimensional scaling (NMDS) analysis demonstrated a significant difference between isolated dystonia patients and HCs (Fig. 1B) (permutational multivariate analysis of variance [PERMANOVA], *R*^2^ = 0.157, *P = *0.002). We observed higher β-diversity in gut microbiota of dystonia patients based on analysis of similarity (ANOSIM), indicating a more heterogeneous community structure among dystonia patients than among HCs (Fig. 1C). Linear discriminant analysis effect size (LEfSe) analysis showed that *Clostridiales* and *Bacteroidetes* were the key taxa distinguishing isolated dystonia patients from HCs. Dystonia patients were significantly enriched in *Blautia*, *Bifidobacterium*, and unidentified *Lachnospiraceae* while depleted in *Bacteroidetes* and unidentified *Clostridiales* in comparison with HCs (Fig. 1D). Further, the relative abundances of the most abundant taxa (≥1%) at different taxonomic levels were further compared (Table S2 and Fig. S2). At the species level, Eubacterium hallii, Blautia obeum, *and* Dorea longicatena were enriched in dystonia subjects, while Bacteroides vulgatus, Bacteroides plebeius, Fusobacterium varium, and Clostridium clostridioforme were enriched in HCs (Fig. 1E).

10.1128/mSphere.00283-21.3 TABLE S2 The significant differences in relative abundance of the most abundant taxa (≥1%) at different taxonomic levels were compared between 16S rRNA gene and metagenomic analyses. The red font denotes the bacteria with significantly higher relative abundance in dystonia patients, while the green font denotes the bacteria with significantly lower relative abundance in dystonia patients. The yellow fill color denotes the significant difference found in both 16S rRNA gene and metagenomic analyses; the light blue fill color denotes the significant difference found only in metagenomic analysis; the light orange fill color denotes the significant difference found only in 16S rRNA analysis. Download Table S2, XLSX file, 0.01 MB. Copyright © 2021 Ma et al. 2021 Ma et al. https://creativecommons.org/licenses/by/4.0/ This content is distributed under the terms of the Creative Commons Attribution 4.0 International license.

10.1128/mSphere.00283-21.8 FIG S2 Average relative abundance comparisons at different taxonomic levels between the isolated dystonia patients and healthy controls revealed by 16S rRNA gene amplicon sequencing. *P* values were determined using the Mann-Whitney U test, and the Benjamini-Hochberg procedure (false discovery rate method correction) was applied to obtain adjusted *P* values for multiple comparisons between groups. Significant differences in taxa (adjusted *P* value < 0.05) are indicated by an asterisk. Boxes indicate 5th to 95th percentiles, with median relative abundances marked as lines and whiskers indicating the range (minimum/maximum) multiplied by the interquartile range (5th to 95th percentiles) from the boxes. Bacterial taxa are ranked by average relative abundances of the overall gut microbiome. Download FIG S2, TIF file, 0.5 MB. Copyright © 2021 Ma et al. 2021 Ma et al. https://creativecommons.org/licenses/by/4.0/ This content is distributed under the terms of the Creative Commons Attribution 4.0 International license.

### Stratification of gut bacterial community in patients with dystonia.

**FIG 2:**
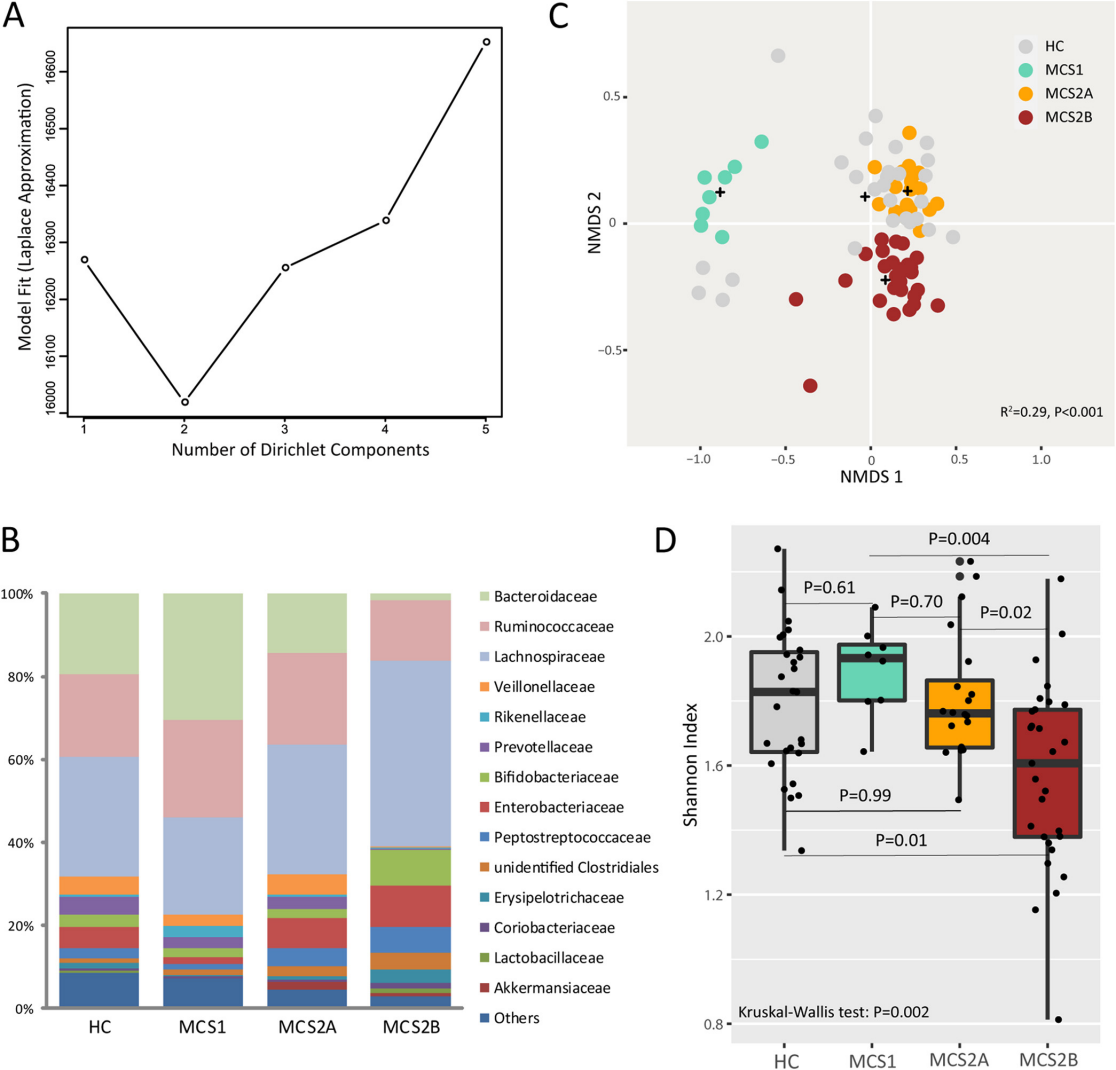
Two compositionally distinct gut microbial states exist in isolated dystonia patients. (A) Based on Laplace approximation, Dirichlet multinomial mixture (DMM) analysis identified two compositionally distinct bacterial communities (*n* = 8 and *n* = 49) in the gut of patients with isolated dystonia. (B) Mean community composition of each microbial state at the family level. (C) NMDS analysis illustrates that DMM-defined gut bacterial communities are compositionally distinct (PERMANOVA, *R*^2^ = 0.29, *P = *0.001). (D) Shannon diversity differs significantly across microbial states (ANOVA, *P* < 0.001).

Given more microbial community heterogeneity, we applied Dirichlet multinomial mixture (DMM) analysis to our cohort to investigate possible gut microbial subgroups in dystonia and identified two significantly distinct microbial community states (MCSs) (*n* = 8 and *n* = 49; *R*^2^ = 0.138; *P* < 0.001) using a Laplace approximation (Fig. 2A). Specific cooccurring bacterial families were characteristically enriched in these groups; the MCS1 (*n* = 8) microbiota was characteristically enriched by *Bacteroidaceae*, *Ruminococcaceae*, and *Lachnospiraceae*. The second larger group, MCS2, which included MCS2A (*n* = 20) and MCS2B (*n* = 29), exhibited a sharply decreased abundance of *Bacteroidaceae*, especially in MCS2B (Fig. 2B). NMDS analysis confirmed a strong and significant relationship among MCS classes and bacterial β-diversity (PERMANOVA, *R*^2^ = 0.29, *P* < 0.001), corroborating the existence of compositionally distinct microbial states (Fig. 2C). These distinct microbial states exhibited significant differences in diversity (Shannon, Kruskal-Wallis tests, *P = *0.002) (Fig. 2D), with MCS2B exhibiting the lowest index. We also investigated whether specific factors (illness duration, age at onset, body mass index [BMI], and severity evaluation scores) were differentially associated with microbial community states. However, no significant correlation was detected.

### Functional analysis of isolated dystonia by metagenomic sequencing.

As shown above, 29 of 57 (51%) of the isolated dystonia patients underwent a gut microbial dysbiosis. To further explore the microbial functional feature of dystonia, we performed metagenomic sequencing of a subgroup consisting of 13 patients from MCS2B and 13 gender- and age-matched HCs. An average of 10.7 ± 2.1 Gb of clean reads was generated per sample, except for one HC sample that was removed due to low depth of sequencing (Table S3). Consistent with the 16S rRNA gene analysis, *B. obeum*, *D. longicatena*, and *E. hallii* were significantly increased in dystonia patients, while B. vulgatus and *B. plebeius* were decreased (Fig. 1F and Fig. S3). In addition, the genera *Alistipes* and *Parabacteroides* were also found to be decreased in patients with dystonia in comparison to HCs (Table S2).

10.1128/mSphere.00283-21.4 TABLE S3 Detailed data information obtained by shotgun metagenomic sequencing for the studied dystonia patients and HCs. Download Table S3, XLSX file, 0.01 MB. Copyright © 2021 Ma et al. 2021 Ma et al. https://creativecommons.org/licenses/by/4.0/ This content is distributed under the terms of the Creative Commons Attribution 4.0 International license.

10.1128/mSphere.00283-21.9 FIG S3 Average relative abundance comparisons at different taxonomic levels between the isolated dystonia patients and healthy controls revealed by shotgun metagenomic sequencing. *P* values were determined using the Mann-Whitney U test, and the Benjamini-Hochberg procedure (false discovery rate method correction) was applied to obtain adjusted *P* values for multiple comparisons between groups. Significant differences in taxa (adjusted *P* value < 0.05) are indicated by an asterisk. Boxes indicate 5th to 95th percentiles, with median relative abundances marked as lines and whiskers indicating the range (minimum/maximum) multiplied by the interquartile range (5th to 95th percentiles) from the boxes. Bacterial taxa are ranked by average relative abundances of the overall gut microbiome. Download FIG S3, TIF file, 0.5 MB. Copyright © 2021 Ma et al. 2021 Ma et al. https://creativecommons.org/licenses/by/4.0/ This content is distributed under the terms of the Creative Commons Attribution 4.0 International license.

**FIG 3:**
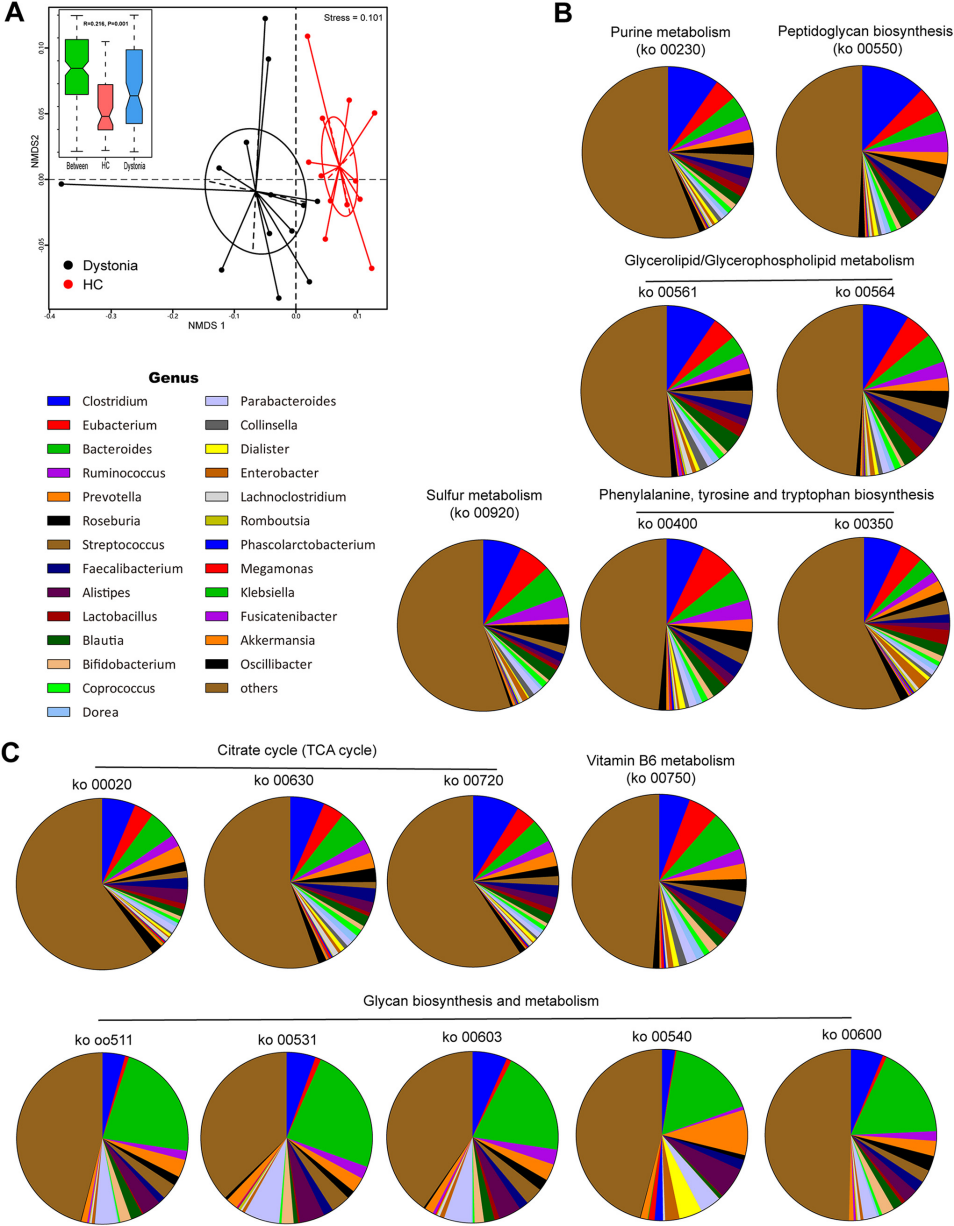
Functional characterization of genes related to dystonia. (A) Functional characterization of the dystonia microbiome. (B and C) NMDS plot of Bray-Curtis resemblance generated from square root-transformed KEGG pathway (level 3) relative abundances. Functional differences in dystonia patients are based on selected metabolic pathways via genus-level composition of 16 modules increased (B) or decreased (C) in isolated dystonia patients compared with HCs.

We further estimated the abundance of metabolic pathways using metagenomic reads mapped to functional orthologs from the KEGG databases to explore differences in the metabolic potential of gut microbiomes between dystonia patients and HCs. Dystonia communities were functionally different from healthy communities and less closely clustered together among individuals, suggesting that interindividual functional variation was higher in dystonia patients than in HCs (Fig. 3A). A total of 22 metabolic pathways (KEGG level 3) were found to be significantly different (*P* < 0.05, false discovery rate [*q*] < 0.02) in abundance (>0.01%) between dystonia patients and HCs, including those involved in nucleotide, amino acid, carbohydrate, and lipid metabolism (Table S4). Interestingly, we identified several different pathways, including phenylalanine, tyrosine, and tryptophan biosynthesis (ko00350, ko00400), purine metabolism (ko00230), and sulfur metabolism (ko00920), which were mainly associated with neurotransmitter’s metabolism, that appeared to be more active in the microbiome of dystonia patients (Fig. 3B). In contrast, genes related to the tricarboxylic acid (TCA) cycle (ko00020, ko00630, ko00720), glycan biosynthesis and metabolism pathways (ko00511, ko00531, ko00540, ko00600, ko00603), and vitamin B_6_ metabolism (ko00750) were less abundant in dystonia patients (Fig. 3C). In addition, we identified an increased gene abundance for acetyl coenzyme A (acetyl-CoA) (an important component in the biogenic synthesis of the neurotransmitter acetylcholine) biosynthesis in dystonia patients compared to controls (system: *trans*-cinnamate degradation, *trans*-cinnamate → acetyl-CoA; phenylacetate degradation, phenylacetate → acetyl-CoA/succinyl-CoA) (Table S4, KEGG module number M0000360, *P* = 0.07, *q* = 0.04). We also traced the contributing genes and determined their likely taxonomic origin to determine which bacteria are involved in these pathways (Fig. 3B and C).

10.1128/mSphere.00283-21.5 TABLE S4 Differences in metabolic potential of gut microbiome between dystonia patients and HCs determined by estimating the abundance of metabolic pathways using our metagenomic reads mapped to functional orthologs from the KEGG databases. Download Table S4, XLSX file, 0.03 MB. Copyright © 2021 Ma et al. 2021 Ma et al. https://creativecommons.org/licenses/by/4.0/ This content is distributed under the terms of the Creative Commons Attribution 4.0 International license.

### Associations between gut microbial species and serum metabolites.

**FIG 4:**
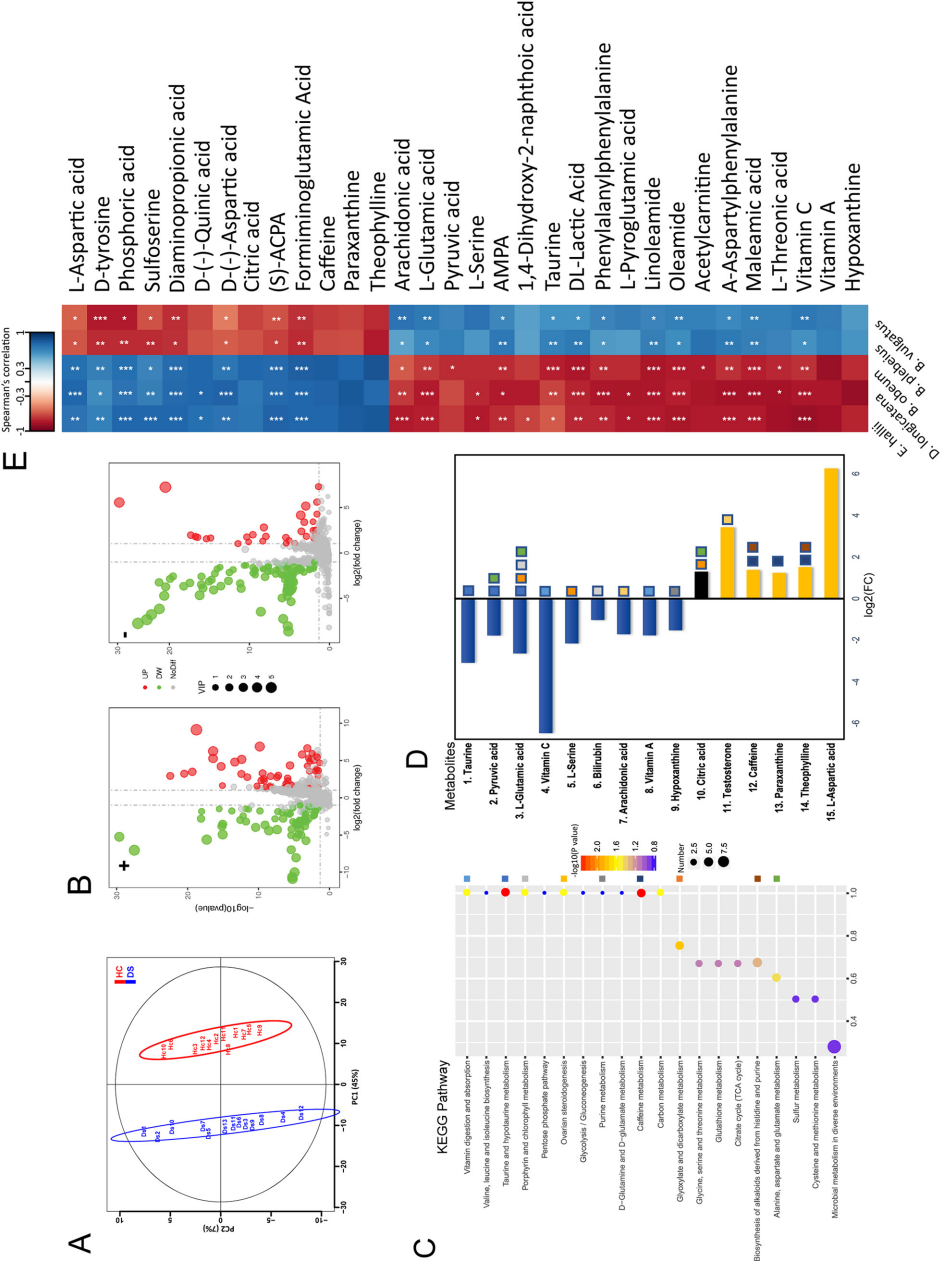
Associations of gut microbial species with circulating metabolites. (A) Supervised partial least-squares discriminant analysis (PLS-DA) showing differences between dystonia patients and HCs. (B) Volcano plot of metabolic features detected in serum samples after background subtraction and removal of the features found in <30% of the data. Positive log_2_ fold change (FC) indicates increased abundance in isolated dystonia patients; +, positive ionization mode; −, negative ionization mode. All *P* values were adjusted using the Bonferroni method. (C) The top 20 pathways of KEGG pathway enrichment. The *x* axis shows the ratio of the number of metabolites in the corresponding pathway to the total number of identified metabolites. The greater the value, the higher the degree of metabolite enrichment in the pathway; the color of the dots represents the (log) *P* value and the size of the dot represents the number of metabolites in the corresponding pathway. The larger number denotes more metabolites in the pathway. The color blocks in the right panel represent different KEGG pathways. The color blocks on the right side were assigned to different KEGG pathways. (D) Significant KEGG pathway enrichment of the significantly changed metabolites. The color blocks in the right panel represent different KEGG pathways. (E) Heatmap of Spearman’s rank correlations between the five bacterial species displaying altered abundance in dystonia patients and 242 different metabolites (only metabolites correlated with at least one species with an adjusted *P* value of <0.01 are shown; *, *P* < 0.05; **, *P* < 0.01; ***, *P* < 0.001).

We performed nontargeted metabolomics profiling of serum samples from the 13 dystonia patients and the 13 HCs in metagenomic analysis and identified 1,543 features defined by retention time and mass/charge ratio, with 224 identified in both positive and negative ionization modes (Table S5). Patients with isolated dystonia showed pronounced metabolic alterations compared with HCs. A supervised partial least-squares discriminant analysis (PLS-DA) using two components (*R*^2^*X* = 0.525, *R*^2^*Y*_cum_ = 0.99, *Q*_cum_^2^ = 0.975, *P* < 0.001) was performed, resulting in some separation tendencies between dystonia cases and HCs (Fig. 4A). Based on the PLS-DA models of metabolite profiling data, 242 metabolites were found to be significantly different in abundance between dystonia cases and HCs, with 82 metabolites having a higher concentration and 160 metabolites having a lower concentration in patients with dystonia (*P* < 0.05; variable importance in projection [VIP] value of >1 and fold change [FC] of >2 or <0.5) (Fig. 4B and Table S5). Among these, 82 metabolites had KEGG identifiers (IDs) in the KEGG database. The dystonia patient serum samples clearly showed the most dramatically decreased levels of several metabolites, including taurine and l-glutamic acid, the most important neurotransmitters, vitamin C, l-serine, ginkgoic acid, aminomethylphosphonic acid (AMPA), and hypoxanthine. In contrast, l-aspartic acid, phosphoric acid, sulfoserine, (*S*)-anti-citrullinated protein antibodies (ACPA), d-tyrosine, and caffeine levels were significantly higher. The VIP scores of these top metabolites were an indication of their contribution to the PLS model (Table S5).

10.1128/mSphere.00283-21.6 TABLE S5 Serum metabolites were profiled by HPLC-MS. Download Table S5, XLSX file, 1 MB. Copyright © 2021 Ma et al. 2021 Ma et al. https://creativecommons.org/licenses/by/4.0/ This content is distributed under the terms of the Creative Commons Attribution 4.0 International license.

To further identify pathways affected by dystonia disease, we performed KEGG pathway enrichment analysis on the 242 significantly different metabolites with known KEGG IDs and identified 14 metabolites (VIP > 1.5, *P* < 0.05) enriched in 9 KEGG pathways: taurine and hypotaurine metabolism, glyoxylate and dicarboxylate metabolism, porphyrin and chlorophyll metabolism, ovarian steroidogenesis, vitamin digestion and absorption, alanine, aspartate, and glutamate metabolism, purine and caffeine metabolism, and biosynthesis of alkaloids derived from histidine and purine (Fig. 4C and D; Table S5). In addition, neurotransmitter-associated metabolites described in the literature, such as d-tyrosine, l-aspartic acid, acetylcarnitine, oleamide, sulfoserine, formiminoglutamic acid, arachidonic acid, and AMPA were also investigated manually. The analyses showed that cell signaling and environmental stress responses were the most relevant biological processes. The most represented metabolic pathways were taurine/hypotaurine, caffeine/purine, and cofactor/vitamin metabolism.

Further, Spearman’s correlation coefficients were computed for relationships between the relative abundance of the five dystonia-associated species identified and the different 242 normalized individual metabolomic features. We identified dystonia-associated gut microbial species linked to changes in serum metabolites. Metabolites were grouped into two clusters depending on the correlations, and correlation coefficients with significant *P* values (<0.01) are shown in Fig. 4E. The abundance of the *Clostridiales* species enriched in patients with dystonia were positively correlated with the first metabolite cluster, including l-aspartic acid, tyrosine, and sulfoserine, and were otherwise negatively correlated with the second cluster, including l-glutamic acid, taurine, pyruvic acid, and vitamin C (Fig. 4E).

Finally, we checked, using the Virtual Metabolic Human database, whether they were made by the species for which we found the most correlations. For l-glutamic acid, taurine, pyruvic acid, l-aspartic acid, tyrosine, and hypoxanthine, we found that the metabolites themselves and their precursors and degradation products had been present in bacteria. No bacterial associations were found for acetylcarnitine, arachidonic acid, and sulfoserine, etc., consequently suggesting that their origin was exclusively human.

## DISCUSSION

Using 16S rRNA gene amplicon and metagenomic sequencing, we investigated gut microbial characteristics in isolated dystonia patients and found that these patients carry an altered gut microbiota enriched with *E. hallii*, *B. obeum*, and *D. longicatena.* Functional analyses also suggested differences in microbial phenylalanine, tyrosine and tryptophan biosynthesis, purine metabolism, and sulfur metabolism pathways. An integrative analysis of the gut microbiome and serum metabolome was employed to gain insight into the links between gut dysbiosis and dystonia, and we found that altered levels of several serum neurotransmitters, especially taurine, l-glutamic acid, and d-tyrosine, were associated with microbial changes, indicating an abnormality in neurotransmitter metabolism in patients with isolated dystonia.

Evidence is accumulating for a potential role of the gut microbiota in the pathogenesis of neurological disorders (7–10). One myoclonic dystonia patient was reported to present a 90% improvement in tremor and abnormal posture after taking anticlostridial antibiotics, hinting at a possible correlation between the pathogenesis of dystonia and the gut microbiome underlying the potential role of *Clostridial* species (10). In this study, intestinal dysbiosis was characterized by significantly increased *Clostridiales* and decreased *Bacteroidetes* in dystonia patients, which might explain the neurological improvement in the above-mentioned patient after taking anticlostridial agents, suggesting that *Clostridiales* may have a potential role in the pathogenesis of dystonia.

*Blautia* and unidentified *Lachnospiraceae*, belonging to the *Lachnospiraceae* family, contributed principally to the increase in *Clostridiales* in dystonia patients. The *Lachnospiraceae* family is known to participate in the breakdown of carbohydrates into SCFAs (11), which are believed to play a key role in microbiota-gut-brain cross talk (12, 13). SCFAs, with acetate, propionate, and butyrate being the most abundant components, may influence the brain by crossing the blood-brain barrier (BBB) (14), modulating neurotransmission, influencing levels of neurotrophic factors, and promoting serotonin (5­hydroxytryptamine [5­HT]) biosynthesis (13). In a cell model, acetate and butyrate promoted serotonin biosynthesis by inducing the expression of tryptophan 5-hydroxylase 1 (the rate-limiting enzyme for 5­HT synthesis) transcription (15). Another study found that butyrate and propionate play a critical role in promoting host 5­HT biosynthesis and in regulating levels of 5­HT in both colon and serum (16). Furthermore, they also induce tyrosine hydroxylase gene transcription (17), which catalyzes the rate­limiting step in the biosynthesis of dopamine, noradrenaline, and adrenaline (18). A decrease in SCFA production can cause intestinal barrier dysfunction, but whether an increased dose of SCFAs could lead directly to adverse effects on locomotor behavior has not been elucidated and requires animal studies in the future. In the present study, the increased abundance of *Clostridiales* might lead to elevated SCFA levels and a consequent increase in 5­HT and dopamine in the gastrointestinal tract.

The abundance of *Bacteroides*, *Parabacteroides*, and *Alistipes* was significantly decreased in isolated dystonia patients in comparison with HCs. A decrease or low level of *Bacteroides* is believed to be associated with metabolic diseases such as obesity (19) and diabetes (20) and psychiatric disorders such as depression (21). Given that no subjects were associated with obesity or diabetes and that there were no differences in body mass index (BMI) between the two groups, an association between *Bacteroides* and metabolic factors can be ruled out in our study. Growing evidence suggests that nonmotor symptoms are essential components of dystonia, with psychiatric comorbidity being most prevalent (22), and a link between dystonia and depression has been confirmed (23). In our cohort, patients were newly diagnosed and there was no statistical difference in Hamilton Depression Scale (HAMD) scores between the two groups, so it is unlikely that depression was a confounding factor in our study. *Alistipes* species are indole positive and may thus influence tryptophan availability (24). Since tryptophan is also the precursor of serotonin, a decreased abundance of *Alistipes* might therefore disrupt the balance of the intestinal serotonergic system.

Until now, the pathophysiological basis of dystonia remains poorly understood. Evidence indicates that the pathophysiological basis of dystonia involves dysfunction of the basal ganglia and cortico-striatal-thalamo-cortical circuits (25), whose activity is modulated among other neurotransmitters by the dopaminergic system (26). In addition to dopamine, a significant neurotransmitter in motor control, serotonin and serotonin-dopamine interactions have received attention (27). Disturbance of the serotonergic system is known to be involved in extrapyramidal movement and psychiatric disorders. Most studies point toward a hypofunction of the serotonergic system in dystonia (27). Analysis of 5-hydroxyindolacetic acid (5-HIAA; the breakdown product of serotonin) in cerebrospinal fluid (CSF) showed significantly reduced levels in patients with idiopathic adult-onset focal dystonia compared with controls (28). A [^11^C]DASB [3-amino-4-(2-dimethylaminomethylphenylsulfanyl)-benzonitrile] positron emission tomography study of cervical dystonia patients revealed a significant correlation between serotonin transporter (SERT) binding in the dorsal raphe nucleus and motor and nonmotor symptoms (29). Higher nondisplaceable binding potential (BPND) was statistically correlated with motor symptom severity, pain, and sleep disturbance, with motor symptom severity being the most important predictor of SERT binding (29). Since *Lachnospiraceae* and *Alistipes* are involved in the production and regulation of 5-HT biosynthesis, the increase in the *Lachnospiraceae* family and the decrease in *Alistipes* species observed in our study may lead to an imbalance in peripheral levels of 5-HT, which might, in turn, modulate brain function by affecting the immune system (30) or signaling to the brain via 5­HT receptors on vagal afferent fibers (31).

Functional analyses revealed that phenylalanine, tyrosine, and tryptophan biosynthesis tend to be more active in dystonia, as reported previously for PD (32). These aromatic amino acids are important as precursors of 5-HT and catecholamines. Correspondingly, metabolome analysis found that the serum concentration of d-tyrosine, the precursor for dopamine, was significantly elevated in dystonia cases. Composition analysis, functional analysis of the gut microbiome, and serum metabolome analysis all point to dopamine and serotonin metabolic disturbance, suggesting that dopaminergic/serotonergic imbalances may be an important modulator of dystonia.

Additionally, the sulfur metabolism pathway appeared to be more active in the microbiome of dystonia patients. In the brain, sulfur metabolism is intimately involved in metabolic cooperation and provides cells with four major reagents critical for methylation reactions (*S*-adenosylmethionine), antioxidant capacity (glutathione), signaling (H_2_S), and cell volume regulation (taurine). An important intermediate in sulfur metabolism is homocysteine (HCY), a neuronal excitotoxic amino acid, and hyperhomocysteinemia is considered an independent vascular risk factor. Increased HCY has also been observed in movement disorders such as PD (33), Huntington’s disease (HD) (34), and dystonia (35, 36). In patients with isolated dystonia, hyperhomocysteinemia was observed independently from immune activation (36). Our study indicates that hyperhomocysteinemia might, to some extent, be related to an increase in sulfur metabolism caused by disturbance of the gut microbiome.

It should be noted that the diversity of the gut microbiome is influenced by several factors, including health status, age, diet, and antibiotics (37). One highlight of our study is the enrollment of drug-naive patients. Evidence is accumulating on the effects that drugs can have on the composition and function of the gut microbiome (38). A case-control study on cause-effect relationships could be performed in the future by analyzing drug-naive dystonia patients. Despite being, to our knowledge, the first study to explore the alterations of gut microbiome in dystonia, the present study has some limitations that should be acknowledged. First, as this is a hospital-based, case-control study, selection bias is unavoidable, and the subjects are not fully representative of the general population. However, our hospital has a big center for neurological diseases and appeals to patients across the country. Patients in our cohort came from 12 provinces and are, to some extent, representative. Second, the cross-sectional design did not allow us to determine the occurrence sequence of gut microbial dysbiosis and dystonia nor to elaborate whether functional abnormalities are directly related to the primary pathophysiology or represent compensatory changes. Third, we are aware that a single case-control study may not be sufficient to fully interpret the relationship between gut microbial dysbiosis and isolated dystonia because of the relatively small number of patients involved. Larger numbers of subjects in longitudinal studies or animal experiments are needed to confirm our findings in the future.

In summary, this is the first study to reveal a significant gut dysbiosis in isolated dystonia patients, which may be associated with altered serum metabolites. Integrative analysis of the microbiome and metabolome suggest that neurotransmitter system dysfunction may be a possible pathway by which the gut microbiome participates in the development of dystonia. The findings provide new insight into the pathogenesis of dystonia, indicating the potential involvement of the enteric neurotransmitter system in dystonic patients. Further validation is needed in animal experiments and in large populations to explore a clearer role of the gut microbiome in the pathogenesis of dystonia.

## MATERIALS AND METHODS

### Participants.

Fifty-seven patients with isolated dystonia (15 male, 42 female) and 27 age- and sex-matched healthy participants (9 male, 18 female) were enrolled. Among the patients, 44 had focal dystonia, 11 had segmental dystonia, and two had general dystonia. All patients, from 12 provinces across China, admitted to the Movement Disorders and Botulinum Toxin-A Treatment center underwent a standardized neurological examination by a specialist in movement disorders. All dystonia patients were newly diagnosed and did not receive oral medicine or botulinum toxin injection. None of the participants had a family history of dystonia. All patients were negative for the genetic testing of *DYT1* (*TOR1A*), *DYT4* (*TUBB4*), *DYT6* (*THAP1*), *DYT23* (*CIZ1*), *DYT24* (*ANO3*), *DYT25* (*GNAL*), and *COL6A3*. Subjects who had no neurological or psychiatric disease were enrolled as healthy controls. Subjects were excluded if they had gastrointestinal diseases, malignant tumors, autoimmune disorders, infectious diseases, or a history of gastrointestinal surgery or had been administered antibiotics or laxatives or immunosuppressive agents in the past 3 months. No subjects were or are on any antianxiety or antidepressant medication. No relevant constipation was present in any of the subjects; dietary and smoking habits did not differ between groups. No detailed dietary plan was requested prior to feces collection, and samples were collected as first bowel movement of the day.

Demographic information (gender, age, age of disease onset, disease duration, height, and weight) and severity evaluation scores, by use of the Global Dystonia Rating Scale (GDS) and the Unified Dystonia Rating Scale (UDRS), were collected from all patients. Evaluation for anxiety and depression, by use of the Hamilton Anxiety Scale (HAMA) and the Hamilton Depression Scale (HAMD), was performed for all subjects. Written informed consent was obtained from all individuals. The study was approved by the ethics committees of Beijing Tiantan Hospital.

### Analysis of fecal microbiota by 16s rRNA gene amplificon sequencing.

Stool samples freshly collected from each participant were immediately transported to the laboratory and frozen at −80°C. Bacterial DNA was extracted, and the V4 region of the 16S rRNA gene was amplified by PCR using barcoded universal fusion primers 515 and 806. PCR products were sequenced and used for taxonomic assignment. The number of reads in each sample was standardized to 37,666 for further analysis. Dirichlet multinomial mixtures (DMM) modeling examines taxon frequencies and determines how many microbial community states (MCSs) exist within a data set by using the DirichletMultinomial (39). See Text S1 in the supplemental material for further details on the laboratory protocols.

10.1128/mSphere.00283-21.1 TEXT S1 Supplemental materials and methods. Download Text S1, DOCX file, 0.02 MB. Copyright © 2021 Ma et al. 2021 Ma et al. https://creativecommons.org/licenses/by/4.0/ This content is distributed under the terms of the Creative Commons Attribution 4.0 International license.

### Functional analysis of microbiome by shotgun metagenomic sequencing.

Total DNA from the 26 fecal samples (13 cases and 13 controls) were subjected to shotgun metagenomic sequencing. Sheared DNA (350 bp) was used for Illumina library construction, and index codes were added to attribute sequences to each sample. Functional annotation was carried out by BLASTP searches against the Kyoto Encyclopedia of Genes and Genomes (KEGG) database (E value of ≤1e−5 and high-scoring segment pair score of >60). For each functional feature (KO in the KEGG database), we estimated its abundance by accumulating the relative abundance of all genes belonging to that feature. See Text S1 for further details.

### Untargeted metabolomics analysis of serum samples.

All serum samples were thawed on ice, and a quality control (QC) sample, made by blending equal volumes (20 μl) of each serum sample, was used to estimate a mean profile representing all the analytes encountered during analysis. Metabolite extraction was performed by mixing 100 μl thawed serum and 400 μl ice-cold 80% methanol and 0.1% formic acid, vortexing the mixture for 30 s, and keeping it at −20°C for 1 h. After centrifugation at 14,000 × *g* and 4°C for 20 min, some of supernatant was diluted to a final concentration containing 53% methanol by liquid chromatography-mass spectrometry (LC-MS)-grade water. The samples were subsequently transferred to a fresh Eppendorf tube and then were centrifuged at 14,000 × *g* at 4°C for 20 min. Finally, the supernatants were subjected to metabolomics profiling by high-performance liquid chromatography (HPLC)-MS in both positive and negative ionization modes. The raw data files were processed using the Compound Discoverer 3.1 (CD3.1; ThermoFisher). Peak intensities were normalized to the total spectral intensity. All data were mean centered and unit variance (UV) scaled before multivariate statistical analysis, and partial least-squares discriminant analysis (PLS-DA) was performed. *R*^2^*X*, *R*^2^*Y*_cum_, and *Q*_cum_^2^ are diagnostics to assess the statistical significance of the model (“cum” = cumulative). *R*^2^*X* and *R*^2^*Y*_cum_ represent the interpretation rate of the model for *X* matrix and *Y* matrix, respectively. The larger the *Q*_cum_^2^, the better the prediction performance of the model. Significantly different metabolites were determined by a combination of a VIP value of >1 from the PLS-DA model and *P* values of <0.05 from two-tailed Student’s *t* tests. See Text S1 for further details.

### Spearman’s multiomics correlation analysis.

Spearman’s correlation coefficients were computed for relationships between the relative abundance of identified dystonia-associated species and normalized individual metabolomic features. A scaled heatmap was constructed for the correlation matrix, including cladogram classification of variables, using the default clustering method. Visual presentation of multiple omics correlations was performed using the Corrplot package in R (v3.5.3). Significant microbiota-metabolite correlations were determined based on an *r* value of less than −0.3 or greater than 0.3 and a false discovery rate (FDR)-adjusted *P* value of <0.05.

### Data availability.

The sequencing data set has been deposited in the Genome Sequence Archive in the BIG Data Center, Beijing Institute of Genomics, Chinese Academy of Sciences, under BioProject accession no. PRJCA003075.

## References

[1] Albanese A, Bhatia K, Bressman SB, Delong MR, Fahn S, Fung VSC, Hallett M, Jankovic J, Jinnah HA, Klein C, Lang AE, Mink JW, Teller JK. 2013. Phenomenology and classification of dystonia: a consensus update. Mov Disord 28:863–873. doi:10.1002/mds.25475. 23649720 PMC3729880

[2] Balint B, Mencacci NE, Valente EM, Pisani A, Rothwell J, Jankovic J, Vidailhet M, Bhatia KP. 2018. Dystonia. Nat Rev Dis Primers 4:25. doi:10.1038/s41572-018-0039-y. 30237473

[3] Lohmann K, Klein C. 2017. Update on the genetics of dystonia. Curr Neurol Neurosci Rep 17:26. doi:10.1007/s11910-017-0735-0. 28283962

[4] Casey DE, Gerlach J, Christensson E. 1980. Dopamine, acetylcholine, and GABA effects in acute dystonia in primates. Psychopharmacology (Berl) 70:83–87. doi:10.1007/BF00432375. 6775341

[5] Jinnah HA, Sun YV. 2019. Dystonia genes and their biological pathways. Neurobiol Dis 129:159–168. doi:10.1016/j.nbd.2019.05.014. 31112762

[6] Benakis C, Martin-Gallausiaux C, Trezzi JP, Melton P, Liesz A, Wilmes P. 2020. The microbiome-gut-brain axis in acute and chronic brain diseases. Curr Opin Neurobiol 61:1–9. doi:10.1016/j.conb.2019.11.009. 31812830

[7] Wood H, Parkinson disease. 2015. Gut reactions—can changes in the intestinal microbiome provide new insights into Parkinson disease. Nat Rev Neurol 11:66. doi:10.1038/nrneurol.2014.256. 25534915

[8] Sun M, Ma K, Wen J, Wang G, Zhang C, Li Q, Bao X, Wang H. 2020. A review of the brain-gut-microbiome axis and the potential role of microbiota in Alzheimer's disease. J Alzheimers Dis 73:849–865. doi:10.3233/JAD-190872. 31884474

[9] Schepici G, Silvestro S, Bramanti P, Mazzon E. 2019. The gut microbiota in multiple sclerosis: an overview of clinical trials. Cell Transplant 28:1507–1527. doi:10.1177/0963689719873890. 31512505 PMC6923550

[10] Borody T, Rosen D, Torres M, Campbell J, Nowak A. 2011. Myoclonus-dystonia affected by GI microbiota. Am J Gastroenterol 106:S351–S352. doi:10.14309/00000434-201110002-00940.

[11] Meehan CJ, Beiko RG. 2014. A phylogenomic view of ecological specialization in the Lachnospiraceae, a family of digestive tract-associated bacteria. Genome Biol Evol 6:703–713. doi:10.1093/gbe/evu050. 24625961 PMC3971600

[12] Silva YP, Bernardi A, Frozza RL. 2020. The role of short-chain fatty acids from gut microbiota in gut-brain communication. Front Endocrinol (Lausanne) 11:25. doi:10.3389/fendo.2020.00025. 32082260 PMC7005631

[13] Dalile B, Van Oudenhove L, Vervliet B, Verbeke K. 2019. The role of short-chain fatty acids in microbiota-gut-brain communication. Nat Rev Gastroenterol Hepatol 16:461–478. doi:10.1038/s41575-019-0157-3. 31123355

[14] Mitchell RW, On NH, Del Bigio MR, Miller DW, Hatch GM. 2011. Fatty acid transport protein expression in human brain and potential role in fatty acid transport across human brain microvessel endothelial cells. J Neurochem 117:735–746. doi:10.1111/j.1471-4159.2011.07245.x. 21395585

[15] Reigstad CS, Salmonson CE, Iii JFR, Szurszewski JH, Linden DR, Sonnenburg JL, Farrugia G, Kashyap PC. 2015. Gut microbes promote colonic serotonin production through an effect of short-chain fatty acids on enterochromaffin cells. FASEB J 29:1395–1403. doi:10.1096/fj.14-259598. 25550456 PMC4396604

[16] Yano JM, Yu K, Donaldson GP, Shastri GG, Ann P, Ma L, Nagler CR, Ismagilov RF, Mazmanian SK, Hsiao EY. 2015. Indigenous bacteria from the gut microbiota regulate host serotonin biosynthesis. Cell 161:264–276. doi:10.1016/j.cell.2015.02.047. 25860609 PMC4393509

[17] Nankova BB, Agarwal R, MacFabe DF, La Gamma EF. 2014. Enteric bacterial metabolites propionic and butyric acid modulate gene expression, including CREB-dependent catecholaminergic neurotransmission, in PC12 cells—possible relevance to autism spectrum disorders. PLoS One 9:e103740. doi:10.1371/journal.pone.0103740. 25170769 PMC4149359

[18] Nagatsu T. 1995. Tyrosine hydroxylase: human isoforms, structure and regulation in physiology and pathology. Essays Biochem 30:15–35. 8822146

[19] Liu R, Hong J, Xu X, Feng Q, Zhang D, Gu Y, Shi J, Zhao S, Liu W, Wang X, Xia H, Liu Z, Cui B, Liang P, Xi L, Jin J, Ying X, Wang X, Zhao X, Li W, Jia H, Lan Z, Li F, Wang R, Sun Y, Yang M, Shen Y, Jie Z, Li J, Chen X, Zhong H, Xie H, Zhang Y, Gu W, Deng X, Shen B, Xu X, Yang H, Xu G, Bi Y, Lai S, Wang J, Qi L, Madsen L, Wang J, Ning G, Kristiansen K, Wang W. 2017. Gut microbiome and serum metabolome alterations in obesity and after weight-loss intervention. Nat Med 23:859–868. doi:10.1038/nm.4358. 28628112

[20] Zhang X, Shen D, Fang Z, Jie Z, Qiu X, Zhang C, Chen Y, Ji L. 2013. Human gut microbiota changes reveal the progression of glucose intolerance. PLoS One 8:e71108. doi:10.1371/journal.pone.0071108. 24013136 PMC3754967

[21] Winter G, Hart RA, Charlesworth R, Sharpley CF. 2018. Gut microbiome and depression: what we know and what we need to know. Rev Neurosci 29:629–643. doi:10.1515/revneuro-2017-0072. 29397391

[22] Zurowski M, McDonald WM, Fox S, Marsh L. 2013. Psychiatric comorbidities in dystonia: emerging concepts. Mov Disord 28:914–920. doi:10.1002/mds.25501. 23893448 PMC3842100

[23] Yang J, Shao N, Song W, Wei Q, Ou R, Wu Y, Shang H-F. 2017. Nonmotor symptoms in primary adult-onset cervical dystonia and blepharospasm. Brain Behav 7:e00592. doi:10.1002/brb3.592. 28239516 PMC5318359

[24] Song Y, Könönen E, Rautio M, Liu C, Bryk A, Eerola E, Finegold SM. 2006. Alistipes onderdonkii sp. nov. and Alistipes shahii sp. nov., of human origin. Int J Syst Evol Microbiol 56:1985–1990. doi:10.1099/ijs.0.64318-0. 16902041

[25] Schirinzi T, Sciamanna G, Mercuri NB, Pisani A. 2018. Dystonia as a network disorder: a concept in evolution. Curr Opin Neurol 31:498–503. doi:10.1097/WCO.0000000000000580. 29746398

[26] Ribot B, Aupy J, Vidailhet M, Mazère J, Pisani A, Bezard E, Guehl D, Burbaud P. 2019. Dystonia and dopamine: from phenomenology to pathophysiology. Prog Neurobiol 182:101678. doi:10.1016/j.pneurobio.2019.101678. 31404592

[27] Smit M, Bartels AL, van Faassen M, Kuiper A, Niezen-Koning KE, Kema IP, Dierckx RA, de Koning TJ, Tijssen MA. 2016. Serotonergic perturbations in dystonia disorders—a systematic review. Neurosci Biobehav Rev 65:264–275. doi:10.1016/j.neubiorev.2016.03.015. 27073048

[28] Naumann M, Götz M, Reiners K, Lange KW, Riederer P. 1996. Neurotransmitters in CSF of idiopathic adult-onset dystonia: reduced 5-HIAA levels as evidence of impaired serotonergic metabolism. J Neural Transmission 103:1083–1091. doi:10.1007/BF01291793. 9013396

[29] Smit M, Vállez García D, de Jong BM. 2018. Relationships between serotonin transporter binding in the raphe nuclei, basal ganglia, and hippocampus with clinical symptoms in cervical dystonia: a [11C]DASB positron emission tomography study. Front Neurol 9:88. doi:10.3389/fneur.2018.00088. 29541052 PMC5835525

[30] Stasi C, Bellini M, Bassotti G, Blandizzi C, Milani S. 2014. Serotonin receptors and their role in the pathophysiology and therapy of irritable bowel syndrome. Tech Coloproctol 18:613–621. doi:10.1007/s10151-013-1106-8. 24425100

[31] Browning KN. 2015. Role of central vagal 5-HT3 receptors in gastrointestinal physiology and pathophysiology. Front Neurosci 9:413. doi:10.3389/fnins.2015.00413. 26578870 PMC4625078

[32] Bedarf JR, Hildebrand F, Coelho LP, Sunagawa S, Bahram M, Goeser F, Bork P, Wüllner U. 2017. Functional implications of microbial and viral gut metagenome changes in early stage L-DOPA-naïve Parkinson's disease patients. Genome Med 9:39. doi:10.1186/s13073-017-0428-y. 28449715 PMC5408370

[33] Saadat P, Ahmadi Ahangar A, Samaei SE, Firozjaie A, Abbaspour F, Khafri S, Khoddami A. 2018. Serum homocysteine level in Parkinson's disease and its association with duration, cardinal manifestation, and severity of disease. Parkinsons Dis 2018:1–6. doi:10.1155/2018/5813084. PMC5954853 29854384

[34] Andrich J, Saft C, Arz A, Schneider B, Agelink MW, Kraus PH, Kuhn W, Müller T. 2004. Hyperhomocysteinaemia in treated patients with Huntington's disease homocysteine in HD. Mov Disord 19:226–228. doi:10.1002/mds.10629. 14978683

[35] Müller T, Woitalla D, Hunsdiek A, Kuhn W. 2000. Elevated plasma levels of homocysteine in dystonia. Acta Neurol Scand 101:388–390. doi:10.1034/j.1600-0404.2000.90339.x. 10877155

[36] Muller UJ, Frick B, Winkler C, Fuchs D, Wenning GK, Poewe W, Mueller J. 2005. Homocysteine and serum markers of immune activation in primary dystonia. Mov Disord 20:1663–1667. doi:10.1002/mds.20667. 16108020

[37] Lozupone CA, Stombaugh JI, Gordon JI, Jansson JK, Knight R. 2012. Diversity, stability and resilience of the human gut microbiota. Nature 489:220–230. doi:10.1038/nature11550. 22972295 PMC3577372

[38] Walsh J, Griffin BT, Clarke G, Hyland NP. 2018. Drug-gut microbiota interactions: implications for neuropharmacology. Br J Pharmacol 175:4415–4429. doi:10.1111/bph.14366. 29782640 PMC6255959

[39] Holmes I, Harris K, Quince C. 2012. Dirichlet multinomial mixtures: generative models for microbial metagenomics. PLoS One 7:e30126. doi:10.1371/journal.pone.0030126. 22319561 PMC3272020

