# Supplementary figures and images for: Gut Microbiome and Serum Metabolome Alterations Associated with Isolated Dystonia
Source: mSphere. 2021 Aug 4;6(4):e00283-21. doi: 10.1128/mSphere.00283-21 (PMC8386414; doi:10.1128/mSphere.00283-21)

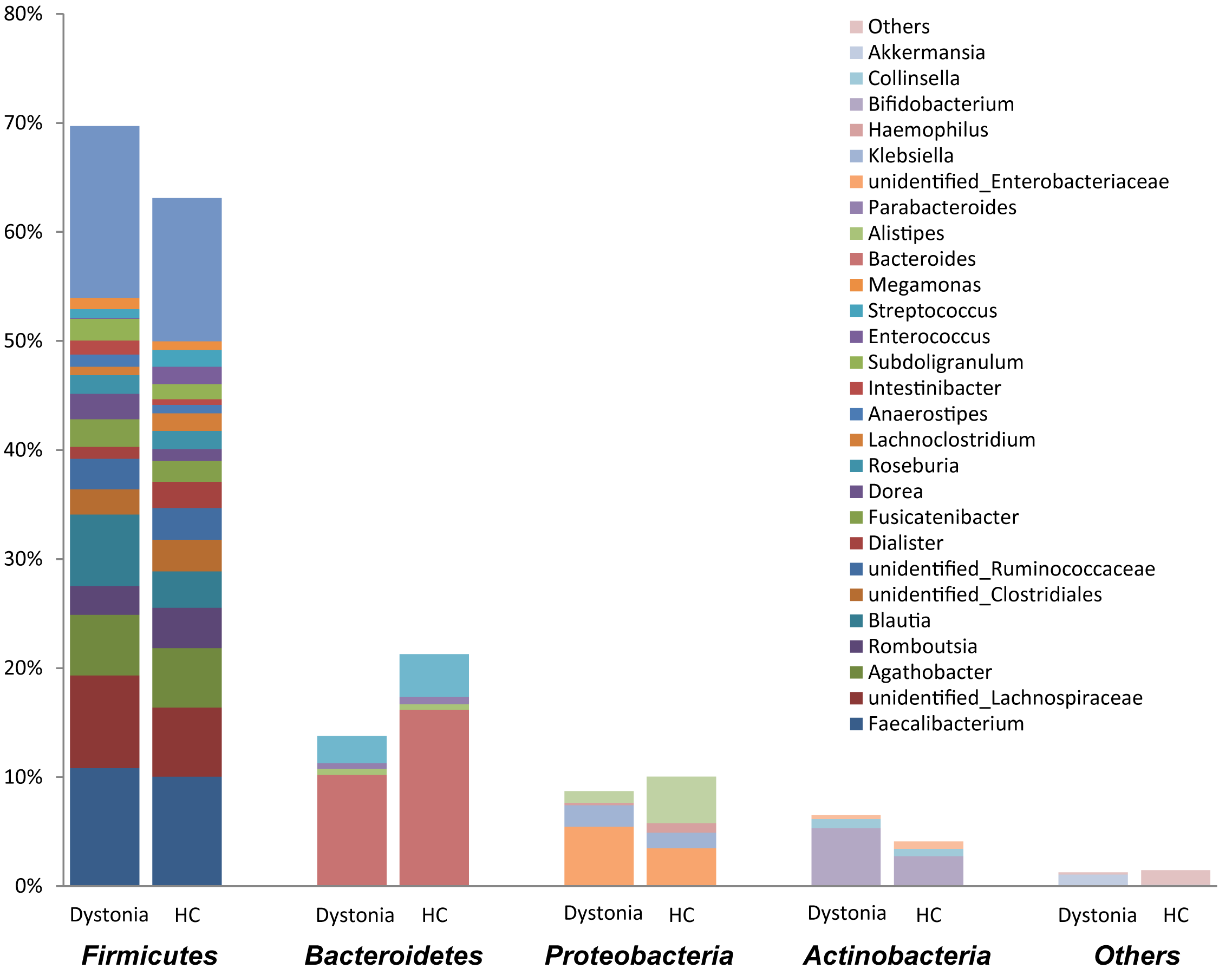

Supplement: FIG S1 [file msphere.00283-21-sf001.tif]

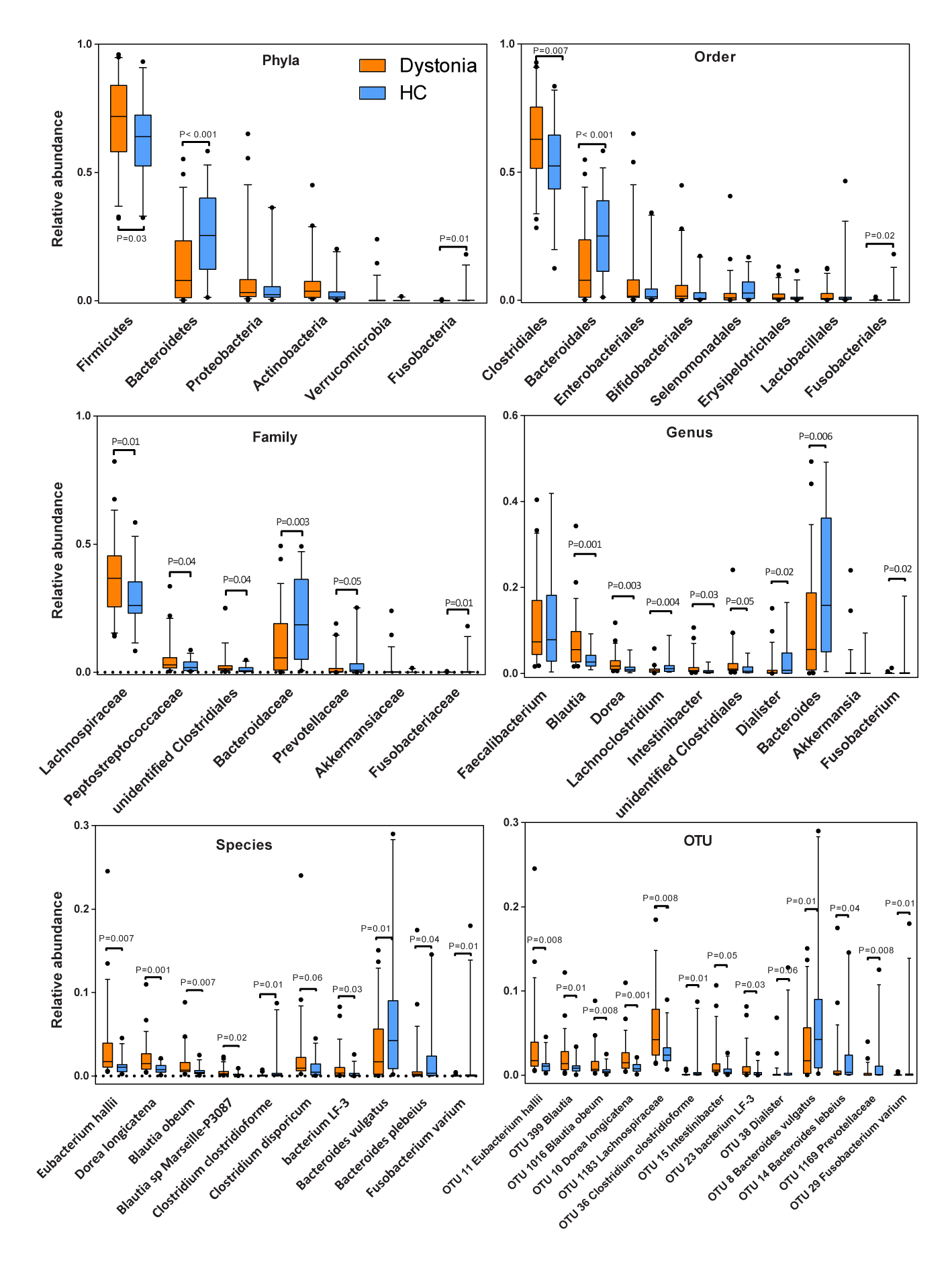

Supplement: FIG S2 [file msphere.00283-21-sf002.tif]

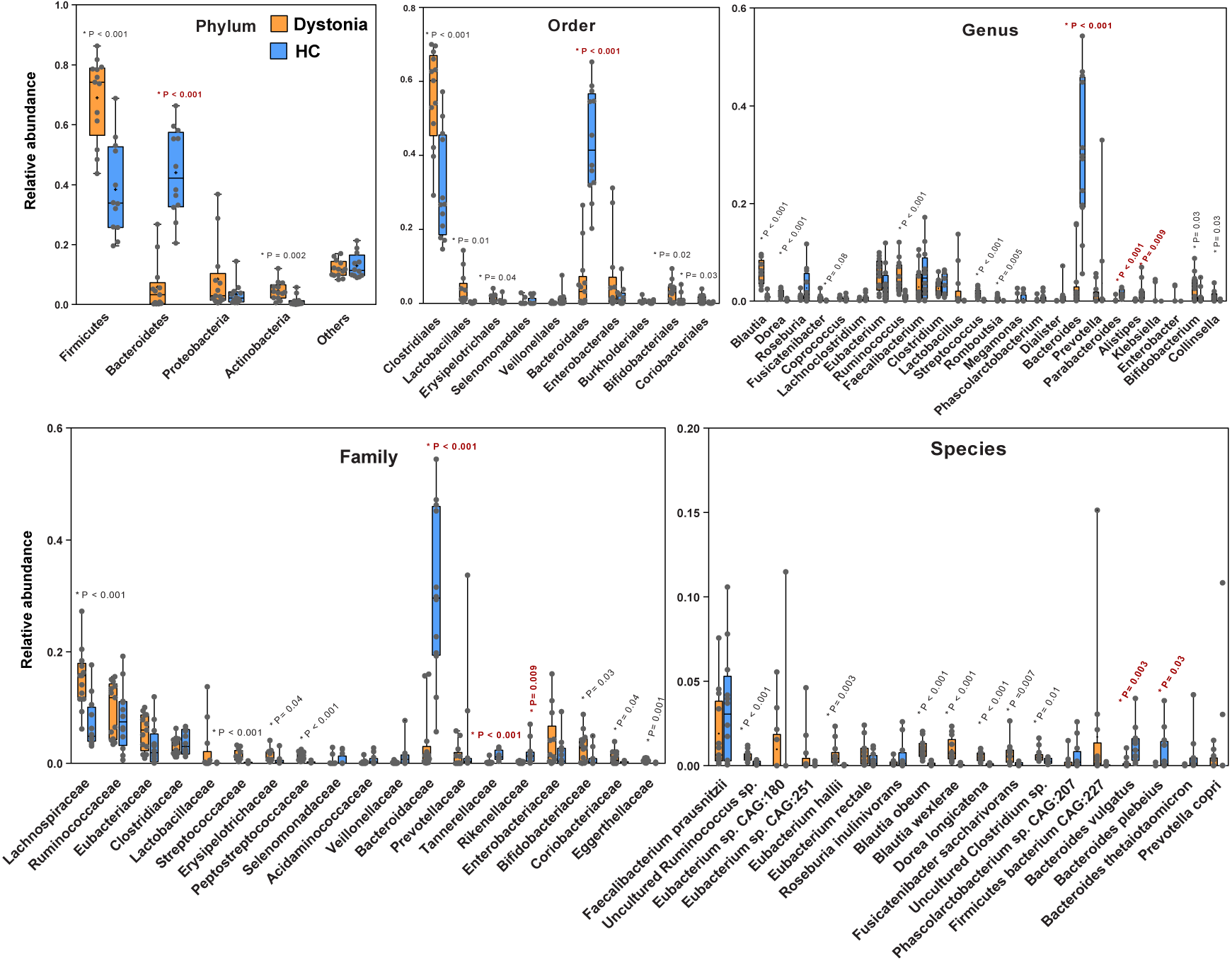

Supplement: FIG S3 [file msphere.00283-21-sf003.tif]
