# Supplementary material for: Gut Microbiome and Serum Metabolome Alterations Associated with Isolated Dystonia
Source: mSphere. 2021 Aug 4;6(4):e00283-21. doi: 10.1128/mSphere.00283-21 (PMC8386414; doi:10.1128/mSphere.00283-21)
Supplement: TEXT S1 [file msphere.00283-21-t0001.docx]

**Supplemental Materials and Methods**

**Participants**

In this study, 57 patients with isolated dystonia (male 15, female 42) and 27 age and sex matched healthy participants (male 9, female 18) were enrolled from Beijing Tiantan Hospital, Capital Medical University. Of the patients, 52 had focal dystonia (44 torticollis, 6 cranial, 2 writer’s cramp), 3 had segmental dystonia and 2 had general dystonia. All outpatients admitted to the Movement Disorders and Botulinum Toxin-A treatment center underwent a standardized neurological examination by a specialist in movement disorders. All the patients are newly diagnosed and did not receive oral medicine or botulinum toxin injection. No family history was reported. Healthy controls (HCs) with normal neurological statue and movement function, absence of neurological or psychiatric disease were selected. Among all the 27 HCs, 19 were relatives of patients who share similar dietary habits. Subjects were excluded if they had gastrointestinal diseases, malignant tumors, autoimmune disorders, infectious diseases, a history of gastrointestinal surgery or were administered antibiotics for more than 3 days in the previous 3 months. Written informed consent was obtained from all individuals. The study was approved by the ethics committees of Beijing Tiantan Hospital.

**Stool Collection and microbial DNA extraction**

Participants were given a stool sampler and provided detailed illustrated instructions for sample collection. Stool samples freshly collected from each participant were immediately transported to the laboratory and frozen at −80 °C immediately. Bacterial DNA in fecal was extracted using QIAamp Fast DNA Stool Mini Kit (Qiagen) and diluted to 1 ng/ml before PCR amplification.

**16S rDNA V4 region amplificon sequencing and data analysis**

The V4 hypervariable region of the 16S rRNA gene was amplified by PCR using barcoded universal fusion primers 515 forward (5’-GTGYCAGCMGCCGCGGTAA-3’) and 806 reverse (5’-GGACTACNVGGGTWTCTAAT-3’). The Agilent Bioanalyzer was used to determine the average size of the amplicon library (389 bp). PCR products were sequenced on a HiSeq 2500 instrument (Illumina, paired end, 2×250). Operational taxonomic units (OTUs) were delineated at the cutoff of 97% using the USEARCH v.8.0. Representative sequences for each OUT were built into a phylogenetic tree by FastTree and subjected to the RDP classifier (RDP database version 11.5, <http://rdp.cme.msu.edu/classifier/classifier.jsp>) to determine the phylogeny with a bootstrap cut-off of 80%. The sequences of all the samples were normalized to 37666 (1000 permutations) to match the difference in sequencing depth. The normalized OUT abundance table was used for the alpha- and beta-diversity analysis. PERMANOVA was used to test for statistical significance between the groups using 9999 permutations. To calculate the variation explained by each of our collected host factors, we performed an Adonis test implemented in R. Dirichlet multinomial mixtures (DMM) was performed to determine how many microbial community states (MCS) exist within a dataset using the DirichletMultinomial. Linear discriminant analysis (LDA) effect size (LEfSe) was performed by coupling standard tests for statistical significance with additional analyses examining biological consistency and effect relevance (LDA score cut-off=2.0) to investigate the features more likely to explain the differences between the dystonia and healthy subjects.

**Shotgun metagenomic sequencing and data processing**

Shotgun metagenomic sequencing was carried out on DNA extracts obtained from the 25 fecal samples (13 cases and 12 controls). For Illumina library preparation, genomic DNA was sheared to a 350-bp average fragment length using a Covaris E210 focused ultrasonicator. Sheared DNA was used for Illumina library construction using the NEBNext® Ultra™ DNA Library Prep Kit for Illumina (NEB, USA) and index codes were added to attribute sequences to each sample. Sequencing libraries were quantified using an Agilent Bioanalyzer 2100. Sequencing was carried out on the Illumina HiSeq x TEN platform (Illumina, San Diego, CA, USA).

Raw Data were preprocessed by using Readfq to acquire the Clean Data for subsequent analysis. Human sequences were subtracted from the dataset using bowtie2 (v2.1.0) and using genomic, mitochondrial, and ribosomal sequences downloaded from NCBI. The Clean Data is assembled and analysed by SOAPdenovo software (V2.04), and then interrupted the assembled Scaftigs from N connection and leave the Scaftigs without N. All samples’ Clean Data are compared to each Scaffold respectively by Bowtie2.2.4 software to acquire the PE reads. All the reads not used in the forward step of all samples are combined and then use the software of SOAPdenovo (V2.04) / MEGAHIT (v1.0.4-beta) for mixed assembly. Filter the fragment shorter than 300 bp in all of Scaftigs for statistical analysis both generated from single or mixed assembly. MetaGeneMark (version 3.26) was used to predict open reading frames (ORFs) in contigs. To obtain a non-redundant gene set, pairwise comparison of predicted ORFs (filtered with a length of 100 bp) was performed using CD-HIT [29] (version 4.5.7) at 95% identity and 90% coverage. The final non-redundant gene catalog contained 1 579 243 microbial genes, which had an average length of 766 bp. DIAMOND software (V0.9.9) was used to blast the Unigenes to the sequences of Bacteria, Fungi, Archaea and Viruses which are all extracted from the NR database. Functional annotations were carried out by BLASTP search against the KEGG database (e value ≤ 1e − 5 and high-scoring segment pair scoring > 60). For each functional feature (KO in KEGG database), we estimated its abundance by accumulating the relative abundance of all genes belonging to this feature.

**Untargeted metabolomics study of serum samples**

All serum samples were thawed on ice and a quality control (QC) sample, made by mixing and blending equal volumes (10 μl) of each serum sample, was used to estimate a mean profile representing all the analytes encountered during analy­sis. We isolated and extracted metabolites (<1,500 Da) as follows. First, 100-μl serum was precipitated with 200-μl methanol, and similarly, the QC sample was precipitated with methanol (1:2 v/v). All samples were subsequently centrifuged at 14,000 g for 10 min at 4 °C. The supernatants were subjected to metabolomics profiling by high performance liquid chromatograph (HPLC)-MS. Sample analysis was performed on Waters ACQUITY ultra-high-performance liquid chromatography system (Milford, MA) coupled with a Waters Q-TOF Micromass system (Manchester, UK) in both positive and negative ionization modes.

The extraction, alignment, deconvolution and further process­ing of raw GC-MS data were referred to the previous published protocols. The final data set was exported as a peak table file, including variables(rt_mz), obser­vations (samples) and peak intensities (abundances). The data was normalized against internal standards. The peak table (named matrix X) file was imported to R software, where PLS-DA were performed. All data were mean-centered and unit variance (UV)-scaled before multivariate statisti­cal analysis. The quality of the models is described by the R2X or R2Y and Q2 values. R2X (PCA) or R2Y (PLS-DA) is defined as the proportion of variance in the data explained by the models and indicates the goodness of fit. Q2 is defined as the proportion of variance in the data predictable by the model and indicates the predictability of current model, calculated by cross-validation procedure. To avoid model over-fitting, a default seven-round cross-validation in SIMCA was performed throughout to determine the optimal number of principal com­ponents. The values of R2X, R2Y, and Q2 were used as indicatives to assess the robustness of a pattern recognition model. The significantly different metabolites were determined by the combination of the VIP value >1 of PLS-DA model and the P values (<0.05) from two-tailed Student’s t test on the normalized peak intensities. Fold change (FC) was calculated as binary logarithm of average nor­malized peak intensity ratio between two groups. The VIP estimates the importance of each variable in the PLS-DA model; variables with a VIP score >1 are important in the model.

**Spearman multi-omic correlation analysis**

Spearman's correlation coefficients were computed for relationships between relative abundances of the identified dystonia-associated species and normalised individual metabolomic features. A scaled heatmap was constructed for the correlation matrix, including cladogram classification of the variables, using the default clustering method. The visual presentation of multiple omics correlations was performed using the Corrplot package in R. Significant microbiota-metabolite correlations were determined based on an r of less than -0.7 or more than 0.7 and an FDR adjusted P value of less than 0.05.
